# Outcomes of atherectomy in treating severely calcified coronary lesions in patients with reduced left ventricular ejection fraction: A systematic review and meta-analysis

**DOI:** 10.3389/fcvm.2022.946027

**Published:** 2022-09-20

**Authors:** Waiel Abusnina, Mostafa Reda Mostafa, Ahmad Al-Abdouh, Qais Radaideh, Mahmoud Ismayl, Mahboob Alam, Jaffer Shah, Noraldeen El Yousfi, Timir K. Paul, Itsik Ben-Dor, Khagendra Dahal

**Affiliations:** ^1^Department of Cardiology, Creighton University School of Medicine, Omaha, NE, United States; ^2^Department of Medicine, Rochester Regional/Unity Hospital, Rochester, NY, United States; ^3^Department of Medicine, University of Kentucky, Lexington, KY, United States; ^4^Section of Cardiology, Department of Medicine, Baylor College of Medicine, Houston, TX, United States; ^5^Medical Research Center, Kateb University, Kabul, Afghanistan; ^6^Tripoli Medical Center, Tripoli, Libya; ^7^Department of Medical Education, University of Tennessee at Nashville, Nashville, TN, United States; ^8^Section of Interventional Cardiology, MedStar Washington Hospital Center, Washington, DC, United States

**Keywords:** reduced ejection fraction, left ventricular dysfunction, severe coronary calcification, atherectomy, percutaneous coronary intervention

## Abstract

**Background:**

Severely calcified coronary lesions with reduced left ventricular (LV) function result in worse outcomes. Atherectomy is used in treating such lesions when technically feasible. However, there is limited data examining the safety and efficacy of atherectomy without hemodynamic support in treating severely calcified coronary lesions in patients with reduced left ventricular ejection fraction (LVEF).

**Objective:**

To evaluate the clinical outcomes of atherectomy in patient with reduced LVEF.

**Methods:**

We searched PubMed, Cochrane CENTRAL Register and ClinicalTrials.gov (inception through July 21, 2021) for studies evaluating the outcomes of atherectomy in patients with severe LV dysfunction. We used random-effect model to calculate risk ratio (RR) with 95% confidence interval (CI). The endpoints were in-hospital and long term all-cause mortality, cardiac death, myocardial infarction (MI), and target vessel revascularization (TVR).

**Results:**

A total of 7 studies consisting of 2,238 unique patients were included in the analysis. The median follow-up duration was 22.4 months. The risk of in-hospital all-cause mortality using atherectomy in patients with severely reduced LVEF compared to the patients with moderate reduced or preserved LVEF was [2.4vs.0.5%; RR:5.28; 95%CI 1.65–16.84; *P* = 0.005], the risk of long term all-cause mortality was [21 vs. 8.8%; RR of 2.84; 95% CI 1.16–6.95; *P* = 0.02]. In-hospital TVR risk was 2.0 vs. 0.6% (RR: 4.15; 95% CI 4.15–15.67; *P* = 0.04) and long-term TVR was [6.0 vs. 9.9%; RR of 0.75; 95% CI 0.39–1.42; *P* = 0.37]. In-hospital MI was [7.1 vs. 5.4%; RR 1.63; 95% CI 0.91–2.93; *P* = 0.10], long-term MI was [7.5 vs. 5.7; RR 1.74; 95%CI 0.95–3.18; *P* = 0.07).

**Conclusion:**

Our meta-analysis suggested that the patients with severely reduced LVEF when using atherectomy devices experienced higher risk of clinical outcomes in the terms of all-cause mortality and cardiac mortality. As we know that the patients with severely reduced LVEF are inherently at increased risk of adverse clinical outcomes, this information should be considered hypothesis generating and utilized while discussing the risks and benefits of atherectomy in such high risk patients. Future studies should focus on the comparison of outcomes of different atherectomy devices in such patients. Adjusting for the inherent mortality risk posed by left ventricular dysfunction may be a strategy while designing a study.

## Introduction

Severely calcified coronary lesions exist in up to 20% of patients undergoing percutaneous coronary intervention (PCI) (1). Performing PCI to severely calcified coronary artery lesions is technically challenging because of the difficulty of balloon or stent delivery and optimal stent apposition and expansion, which may lead to underexpansion with increased risk of stent thrombosis and restenosis (2). Atherectomy is one of the effective ways to treat severely calcified coronary lesions that can modify calcified plaques to facilitate balloon or stent delivery and optimize stent expansion (3, 4). The 2011 American College of Cardiology Foundation/American Heart Association/Society for Cardiovascular Angiography and Interventions PCI guidelines gave Class IIa (level of evidence C) recommendation for rotablator for the treatment of heavily calcified plaques that cannot be crossed by a balloon catheter or adequately dilated before stenting (5).

Patients with severe coronary artery calcifications and severely reduced left ventricular ejection fraction (LVEF) have worse prognosis than those with normal LVEF, and pose technical challenges in standard PCI techniques (6). Although coronary artery bypass grafting (CABG) has been recommended for patients with left ventricular (LV) systolic dysfunction as a first line revascularization strategy (6), due to the technological advances and improvements in intervention techniques, high-risk PCI using atherectomy is an alternative to high-risk CABG (7–9). As there are a few reports that have focused on the clinical outcomes of atherectomy in patients with severe LV dysfunction, we aimed to perform a systematic review and meta-analysis of available literature in this regard.

## Methods

### Data source and search strategy

A meta-analysis was performed according to the Preferred Reporting Items for Systematic Reviews an Meta-Analyses (PRISMA) 2009 guidelines (10). Two reviewers (WA and MA) independently identified the relevant studies by an electronic search of the PubMed, EMBASE, Cochrane Central Register of Controlled Trials, and ClinicalTrials.gov databases (from inception to July 2021) (Supplementary Table 1). References of the retrieved studies were also screened further for relevant studies. The following search terms and key words were used: “atherectomy” OR “orbital atherectomy” OR “rotational atherectomy” AND “reduced ejection fraction” OR “heart failure with reduced ejection fraction” OR “left ventricular dysfunction” AND “severely calcified coronary lesions” and “coronary artery disease.” There was no language, publication date or publication status restrictions imposed.

### Study selection

Two investigators (WA and MA) independently assessed the eligibility of studies on the basis of titles, abstracts, and full-text reports. Discrepancies in study selection were discussed and resolved with the third investigator (KD). Eligible studies had to satisfy the following prespecified criteria: (a) Studies evaluating the use of atherectomy for coronary lesions in patients with left ventricular dysfunction (b) availability of clinical outcome data. The exclusion criteria were lack of any clinical outcome data and duplicated publications. Our study population included patients with severely reduced LVEF, moderate LVEF and preserved LVEF. We compared patients with severely reduced LVEF as one groups to the combined (moderate and preserved LVEF) as one group. Severely reduced LVEF and moderate LVEF were attributed according to the definition used in each study as shown in (Table 1).

**Table 1:** Study characteristics included in the meta-analysis.

**Study**	**Type**	**Total**	**Type of Atherectomy**	**# of arms**	**Severely reduced EF**	**Moderate reduced EF**	**normal EF**	**Follow up**
Lee et al. (8)	prospective	437	orbital	three groups	26–40%	41–50%	> 50%	In-hospital/12 months
Shlofmitz et al. (15)	retrospective	438	orbital	two groups	≤ 40	>40	30 days
Watanabe et al., (11)	retrospective	270	rotational	two groups	≤ 35%	>35%		in–hospital/30 days
Whiteside et al., (12)	retrospective	131	rotational	three groups	≤ 30 %	30–50%	>50%	in-hospital
Mankerious et al. (13)	retrospective	644	rotational	three groups	≤ 35%	36–54%	≥55%	in-hospital/5 years
Zhang et al. (3)	retrospective	140	rotational	three groups	≤ 35%	36–50%	>50%	in-hospital/24 months
Yoshida et al. (14)	retrospective	178	rotational	three groups	≤ 35%	36–50%	>50%	in-hospital/12 months

EF, ejection fraction.

### Data extraction and quality assessment

Two investigators (WA and QA) independently extracted data (baseline characteristics, definition of outcomes and number of events) using a standardized data abstraction form. The same investigators independently and systematically assessed the studies' methodological quality using the Newcastle Ottawa Scale (Supplementary Table 2), disagreements were resolved by a third author (KD). We assessed for publication bias using the funnel plots for the outcomes (Supplementary Figure 1).

### Outcome measures

The endpoints were in-hospital and long term all-cause mortality, cardiac death, myocardial infarction (MI), and target vessel revascularization (TVR). Procedural outcomes were also analyzed including slow or no reflow, perforation and dissection. Endpoints were attributed according to the definition used in each study. Definition of MI in each included study were reported in the Supplementary Table 3.

### Statistical analysis

For categorical outcomes, the risk ratios (RRs) with 95% confidence intervals (CI) were calculated and study -specific RR were combined with the DerSimonian and Laird random effects model with the estimate of heterogeneity using the Mantel–Haenszel model. We used I^2^ statistic to measure heterogeneity among the trials; a value of 0% indicates no observed heterogeneity, and values of 25, 50, and 75% define low, moderate, and high heterogeneity, respectively. The presence of publication bias for each outcome was investigated by visual estimation of funnel plots when data was available for at least three studies. Results were reported according to the Preferred Reporting Items for Systematic Reviews and Meta-Analyses Protocol (PRISMA-P) 2015 statement. Analyses were performed using Review Manager (RevMan) Version 5.3 (The Nordic Cochrane Center, The Cochrane Collaboration, Copenhagen, Denmark).

## Results

### Search results

**Figure 1:**
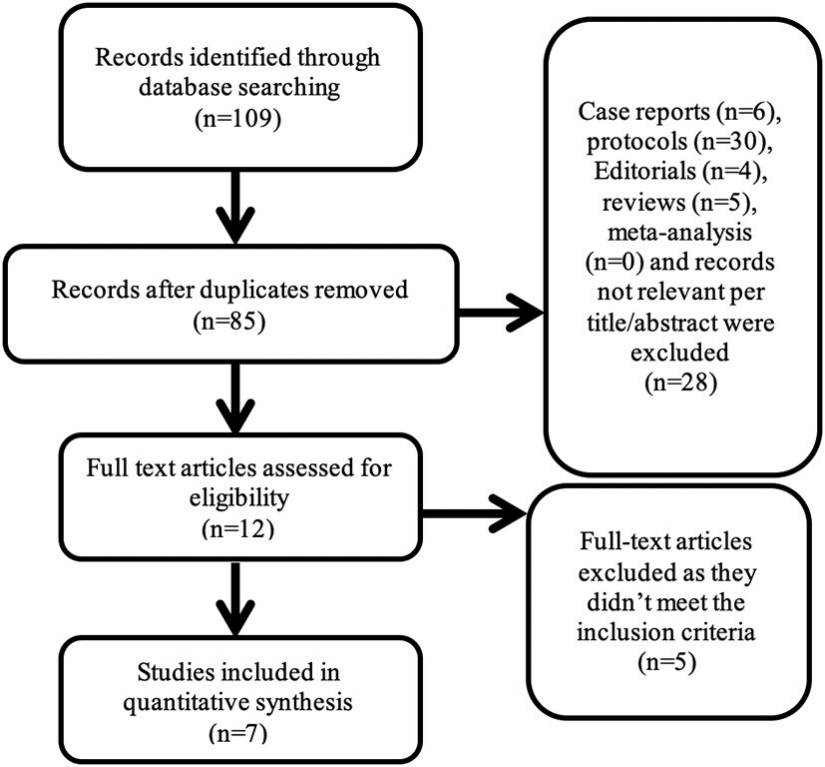
PRISMA flow diagram for study selection.

Figure 1 displays the PRISMA diagram for study search and selection. A total of seven studies (six retrospective and one prospective studies) including 2, 238 patients, 270 with severely reduced LVEF, and 1,968 patients with moderately reduced or preserved LVEF were included in the meta-analysis. The median follow-up duration for long term outcome was 22.4 months. The characteristics of the included studies and the patients' demographics are presented in Tables 1, 2, respectively.

**Table 2:** Baseline characteristics of the all the included studies.

**Study**	**Lee Al 2017**			**Shlofmitz et al**. **(****15****)**		**Watanabe et al**. **(****11****)**		**Whiteside et al**. **(****12****)**			**Mankerious et al**. **(****13****)**			**Zhang et al**. **(****3****)**			**Yoshida et al**.^**3**^ **(****14****)**		
	**EF (26-40%)**	**EF 41–50%**	**EF > 50%**	**EF ≤ 40**	**EF > 40**	**EF ≤ 35%**	**EF > 35%**	**EF ≤ 3 0**	**EF 30–50**	**EF > 50**	**EF ≤ 35%**	**EF 36–54**	**EF ≥ 55**	**EF ≤ 35%**	**EF (36-50%)**	**EF>50%**	**EF ≤ 35%**	**EF 36-50%**	**EF ≥50**
Number of participants	33	90	314	69	369	33	237	18	42	71	82	170	392	10	11	119	25	44	109
Age, mean (years)	71.3 ± 1.9	71.7 ± 1.1	71.3 ± 0.5	73.8 ± 12.3	73.6 ± 9.5	69.7± 7	70.8 ± 8.8	69.6 ± 11.7	66.6 ± 11.2	67.9 ± 8.9	72.5 ± 8.7	72.9 ± 8.3	71.9 ± 8.4	76 ± 9.3	75.9 ± 9.2	69.9 ± 9.1	72.2 ± 8.8	76.8 ± 8.6	73.9 ± 8.2
Men	84.8%	75.6%	59.9%	71%	67.5%	76%	75.9%	77.8%	76.2%	60.6%	78%	75.3%	73.5%	40%	45.5%	60.5%	60%	77.3%	59.6%
Diabetes mellitus	42.4%	46.7%	33.1%	50.7%	39.8%	55%	51.3%	61.1%	54.8%	53.5%	32.1%	37.6%	34.1%	70%	54.5%	49.6%	52%	50%	62.4%
Previous Myocardial infarction	53.1%	37.5%	15.4%	31.9%	12.7%	91%	28.7%	72.2%	38.1%	15.5%	N/A	N/A	N/A	40%	36.4%	15.1%	20%	38.6%	16.5%
Hypertension	90.9%	91.1%	91.7%	82.6%	86.7%	88%	88.9%	100%	95.2%	85.9%	90.1%	94.7%	89.5%	100%	63.6%	75.6%	28%	43%	64%
Chronic kidney disease	N/A	N/A	N/A	36.8%	15.8%	90%	54.9%	n/a	N/A	N/A	32.2%	24.4%	11%	n/a	n/a	n/a	72%%	45.5%	47.7%
Smoker	75.8%	74.4%	62.7%	11.6%	3.0%	58%	63.9%	n/a	N/A	N/A	33.3%	27.6	32.6%	n/a	n/a	n/a	60%%	75.1%	50.2%
Imeplla	N/A	N/A	N/A	9 (13%)	0	N/A	N/A	N/A	N/A	N/A	1 (1.2%)	0	0	N/A	N/A	N/A	N/A	N/A	N/A
IABP	N/A	N/A	N/A	6 (8.7%)	6 (1.6)	5 (15%)	5 (2.1%)	2 (11.1%)	1 (2.4%)	0	4 (4.9%)	1 (0.6%)	1 (0.3%)	N/A	N/A	N/A	7 (24.1%)	2 (4.4%)	13 (11%)
ECMO	N/A	N/A	N/A	1 (1.4%)	0	N/A	N/A	N/A	N/A	N/A	N/A	N/A	N/A	N/A	N/A	N/A	N/A	N/A	N/A

EF, ejection fraction; N/A, not available.

### Clinical outcomes

**Figure 2:**
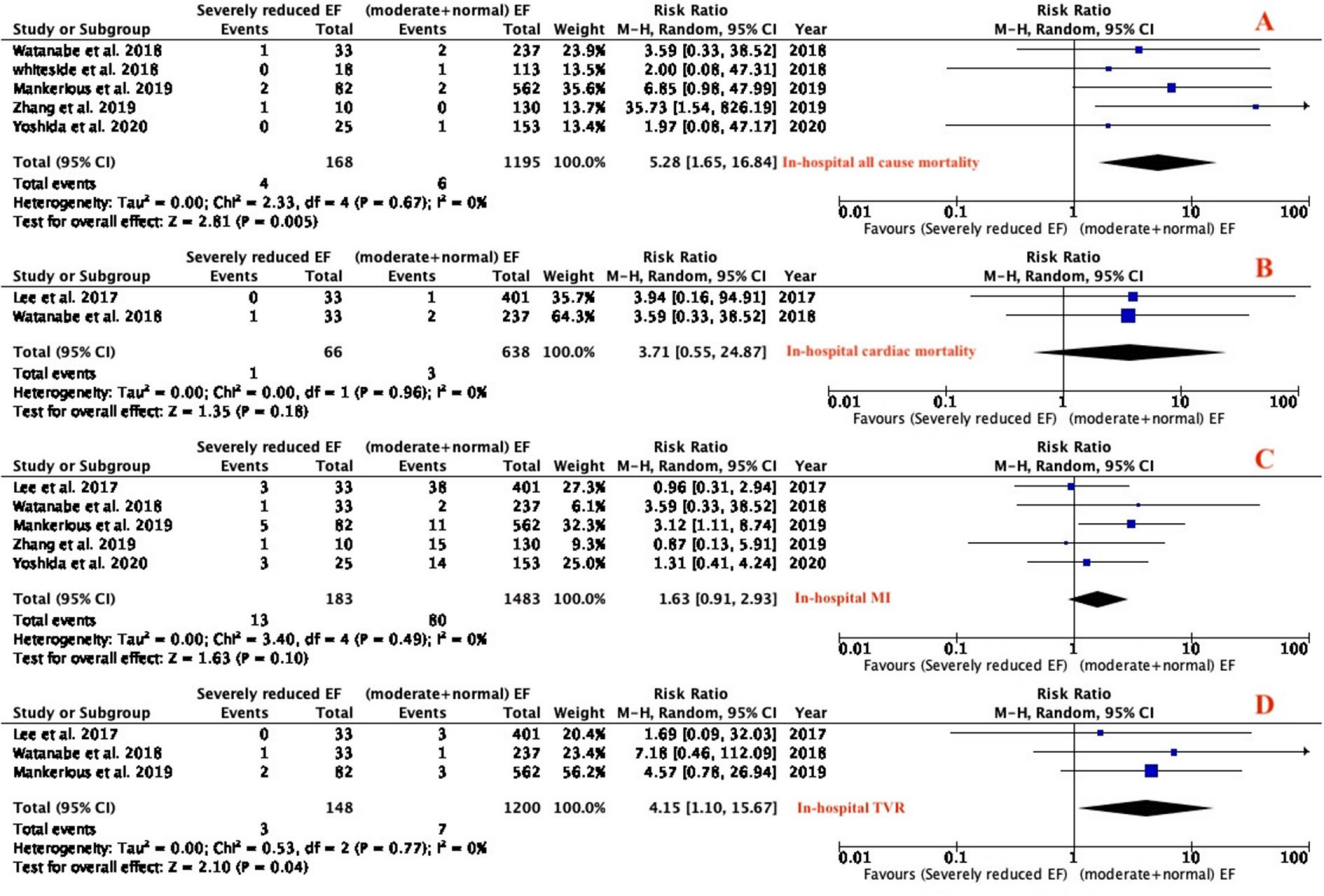
Forest plot of in-hospital outcomes (all-cause mortality, cardiac mortality, MI, TVR). (MI, myocardial infarction; TVR, target vessel revascularization).

**Figure 3:**
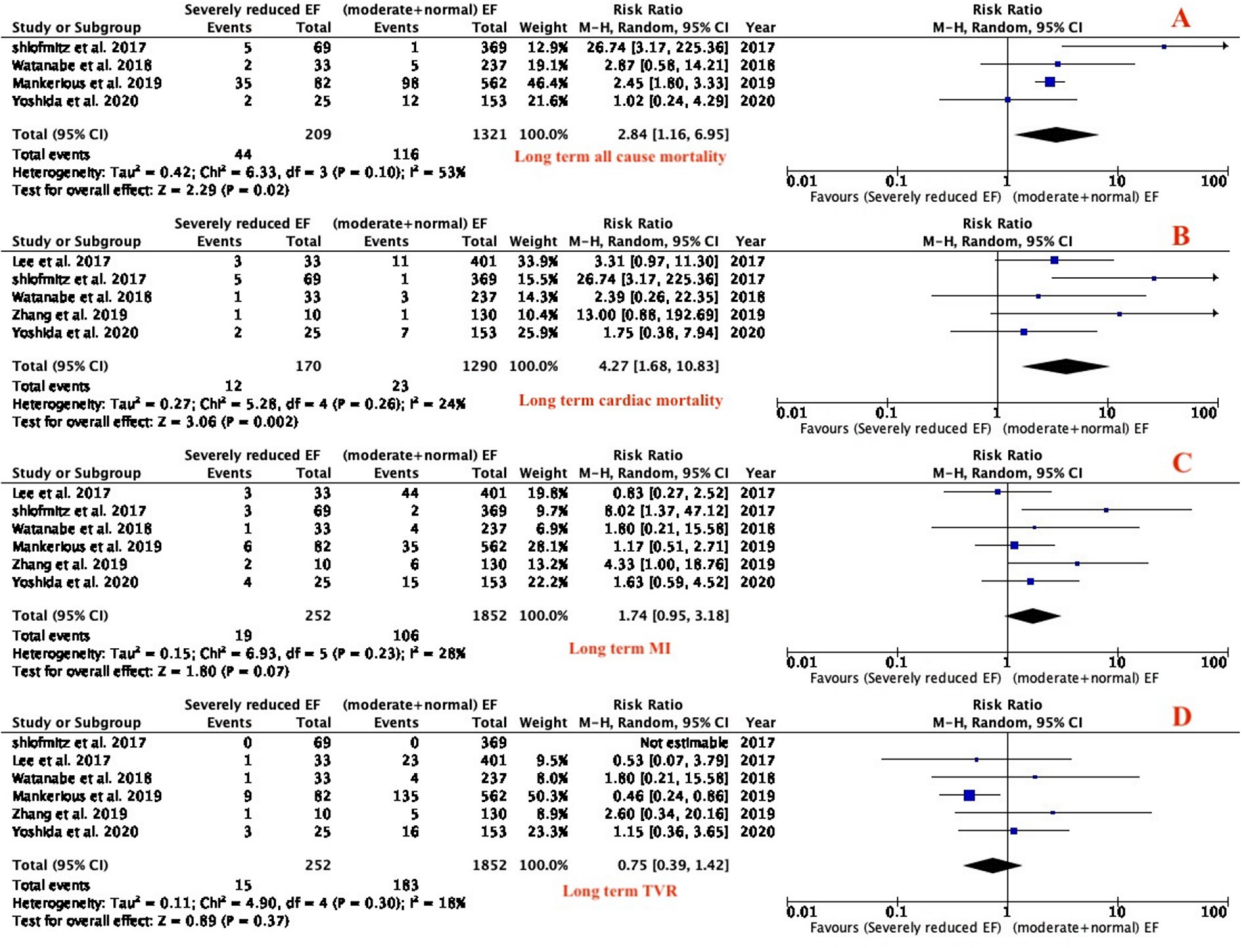
Forest plot of long term outcomes (all-cause mortality, cardiac mortality, MI, TVR). (MI, myocardial infarction; TVR, target vessel revascularization).

In-hospital and long term outcomes (all-cause mortality, cardiac death, MI and TVR) were reported in all the studies. In-hospital all-cause mortality and TVR were significantly higher in patients with severely reduced LVEF as compared to patients with moderate and preserved LVEF who had atherectomy (RR: 5.28; 95% CI 1.65–16.84; *P* = 0.005; I^2^ = 0%; Figure 2A) and (RR: 4.15; 95% CI 4.15–15.67; *P* = 0.04; I^2^ = 0%; Figure 2D), respectively. There was no significant difference between the two groups in in-hospital cardiac death (RR of 3.71; 95% CI 0.55–24.87; *P* = 0.18; I^2^ = 0%; Figure 2B) and MI (RR of 1.63; 95% CI 0.91–2.93; *P* = 0.10; I^2^ = 0%; Figure 2C). Patients with severely reduced LVEF had higher rate of long term all-cause mortality (RR of 2.84; 95% CI 1.16–6.95; *P* = 0.02; I^2^ = 53%; Figure 3A) and long-term cardiac death (RR of 4.27; 95% CI 1.68–10.83; *P* = 0.002; I^2^ = 24%; Figure 3B) compared with moderate and preserved LVEF. There was no difference between the two groups in term of long-term MI (RR of 1.74; 95% CI 0.95–3.18; *P* = 0.07; I^2^ = 28%; Figure 3C) and long-term TVR (RR of 0.75; 95% CI 0.39–1.42; *P* = 0.37; I^2^ = 18%; Figure 3D).

### Procedural complications

**Figure 4:**
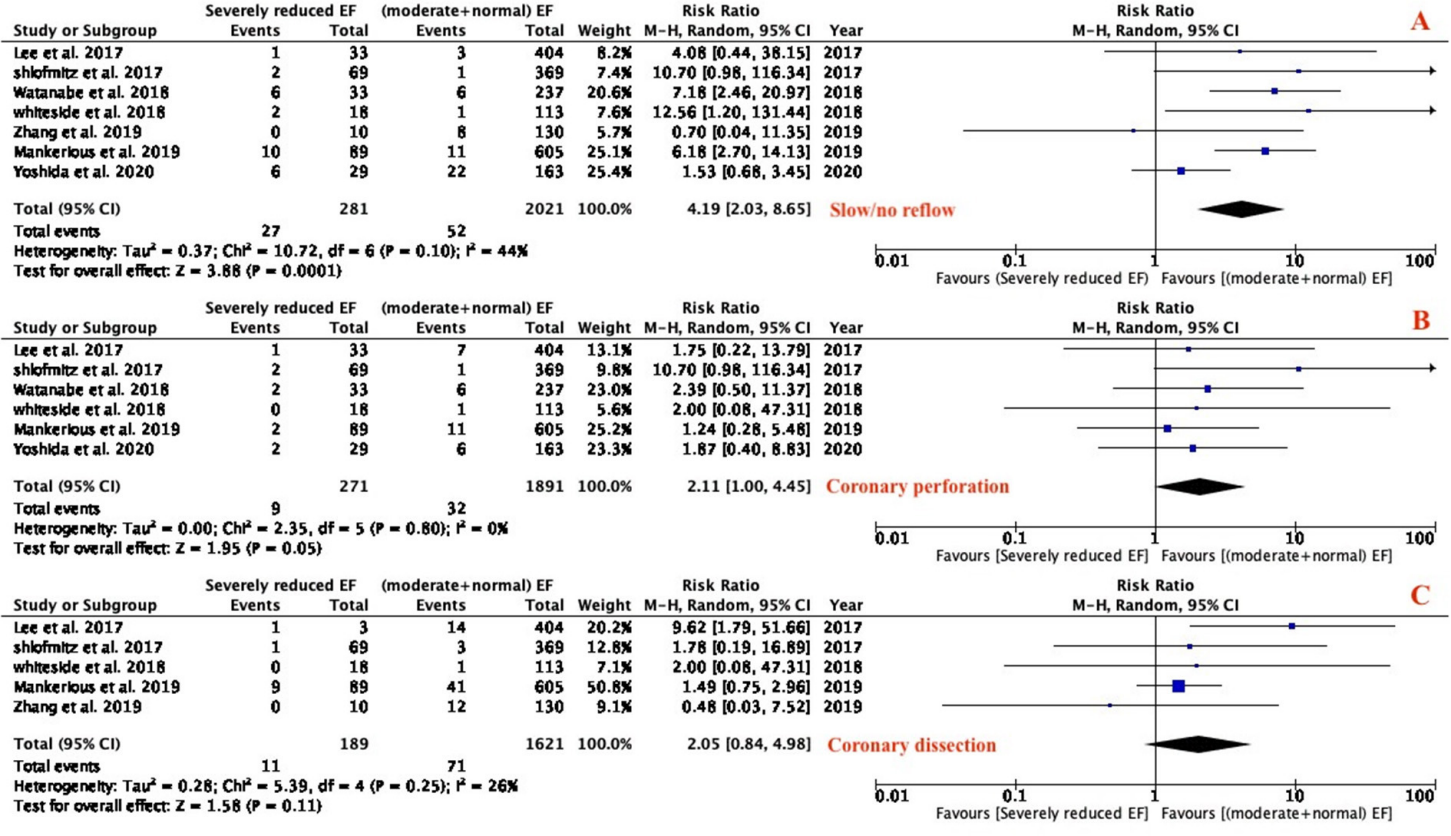
Forest plot of procedural outcomes (slow/no reflow, coronary perforation, dissection).

Procedural complications including slow flow or no reflow, coronary perforation, and coronary dissection were analyzed (Figure 4). Slow or no reflow was significantly higher in patients with severely reduced LVEF (RR: 4.19; 95% CI 2.03–8.65; *P* = 0.0001; I^2^ = 44%; Figure 4A). Atherectomy in patients with severely reduced LVEF is associated with higher coronary perforation (RR: 2.11; 95% CI 1.00–4.45; *P* = 0.05; I^2^ = 0%; Figure 4B). There was no difference between the two groups in peri-procedural dissection (RR: 2.05; 95% CI 0.84–4.98; *P* = 0.11; I^2^ = 26%; Figure 4C).

### Heterogenicity, publication bias and asymmetry

With respect to clinical outcomes, there was no heterogeneity for in-hospital all-cause mortality (*P* = 0.005, I^2^ = 0%), cardiac mortality (*P* = 0.18, I^2^ = 0%), MI (*P* < 0.10, I^2^ = 0%) and TVR (*p* < 0.04, I^2^ = 0%). Heterogenicity was low for long term cardiac mortality (*P* < 0.002, I^2^ = 24%), MI (*P* < 0.07, I^2^ = 28%), and TVR (*P* < 0.37, I^2^ = 18%). There was moderate heterogeneity for long term all-cause mortality (*P* = 0.02, I^2^ = 53%). Overall, heterogenicity was low and there was no evidence of publication bias on visual inspection of funnel plots for the clinical outcomes (Supplemental Figure 1).

## Discussion

The current meta-analysis evaluated in-hospital and long-term outcomes of patients with severely calcified coronary lesions who underwent PCI with atherectomy. We divided the patients in two groups depending on their LV dysfunction and compared their clinical risks. The main findings of the analysis were (1) Treating severely calcified coronary artery disease with atherectomy in patients with severely reduced LVEF had significant higher in-hospital and long term all-cause mortality risks compared to the patients with moderate or preserved LVEF, (2) There was no significant difference in in-hospital cardiac mortality between the two groups while long term cardiac mortality was significantly higher in patients with severe coronary artery calcification (CAC) and severely reduced LVEF who underwent atherectomy, (3) The risk of in-hospital MI was higher in patients with severely reduced LVEF while no difference in long term MI compared to those patients with moderately reduced or preserved LVEF. (4) There was increased risk of no reflow/slow flow but similar risk of coronary perforation and dissection between two groups.

Severe CAC makes PCI challenging and difficult to achieve optimal results. Despite advances in interventional equipment and techniques, the outcome in patients with severe CAC remains worse than those with non-calcified coronary stenosis (16, 17). Generally, PCI in patients with LVEF <35% is associated with higher in-hospital mortality rates (18) and is considered as a high-risk PCI (5, 6). Patients with severe CAC and left ventricular systolic dysfunction undergoing PCI has worse prognosis and increased risk of adverse events after PCI, including death (19–21). Left ventricular systolic dysfunction was also reported to be an independent predictor of poor clinical outcomes in patients undergoing rotational atherectomy (22). The atherectomy technique has been widely critiqued for its association with high complication rates, and is still performed in patients with high pre-procedural risk, like patients with severely LVEF, which can further increase the risk(23). Pivotal Trial to Evaluate the Safety and Efficacy of the Diamondback 360^°®^ Orbital Atherectomy System in Treating De Novo, Severely Calcified Coronary Lesions (ORBIT II) showed that preparation of severely calcified plaque with the Orbital Atherectomy System not only helped facilitate stent delivery, but had lower rates of in-hospital Q-wave MI (0.7%), cardiac death (0.2%), and TVR(0.7%).(24) In addition, the rate of 1-year cardiac death in ORBIT II study increased as LVEF decreased. However, a study by Jujo et al. (25) suggested that using rational atherectomy in treating treat severely calcified coronary artery stenoses as therapeutic strategy, is associated with increased rates of PCI success and improved long-term CV mortality.

There are several explanations for the increased mortality among severely reduced LVEF patients with atherectomy. First, there is inherently increased mortality associated with low LVEF compared to normal EF patients (26) Second, the use of atherectomy can increase the risk of the mechanical and thermal injury and distal micro embolization causing no-reflow or slow flow, which may carry a more deleterious effect among low LVEF than preserved LVEF patients (27) with the resulting microvascular dysfunction leading to worse outcomes. An analysis from National Cardiovascular Data Registry CathPCI Registry showed increase in the temporal use of coronary atherectomy, either orbital or rotational, with a temporal decline in MI (odds ratio 0.97 [95%CI 0.96–0.98], *P* < 0.001) (4). On the other hand, McEntegart et al. (28) reported that rotational atherectomy was associated with a type 4A MI rate of 24% as detected by cardiac magnetic resonance imaging. Our study demonstrated non-significant difference in both in-hospital MI [(RR of 1.63; 95% CI 0.91–2.93; *P* = 0.10) and long-term MI (RR of 1.74; 95% CI 0.95–3.18; *P* = 0.07] in patients with severely reduced LVEF compared to the other group. However, Atherectomy has been tested in several high risk population and showed comparable adverse cardiac events in patients with acute coronary syndrome and in patients with calcified lesions ≥25 mm of length (29, 30). While atherectomy in reduced EF was independently linked to worse cardiac events including death and MI (22, 31), PROTECT II (32) has shown that the use of mechanical support device during atherectomy was associated with poor MACE in reduced EF groups which denotes that the utility of mechanical support devices would be an independent predictor of worse outcomes in such a compromised subset of population.

Our meta-analysis demonstrates higher rates of in-hospital TVR in the groups with severely reduced LVEF while the long term TVR rates were not different between the two groups. One study showed that TVR rates at 5 years were not affected by LVEF (13). In ORBIT II, the rate of 1-year TVR was comparable across the LVEF groups (24). The lack of difference in TVR rates at follow up between the compared groups might be explained by the late catch-up phenomena, and the likelihood of microembolization having more deleterious effect in patients with depressed LVEF. It was also reported that in severely calcified lesions that necessitated rotational atherectomy, thin-strut drug eluting stent resulted in lower rates of TVR compared to thick-strut drug eluting stent (33).

## Limitations

The main limitation of this meta-analysis is that the cohort of patients with severely reduced LVEF are inherently high risk population with worse outcomes due to underlying cardiac pathology independently from the atherectomy use. Another major limitation of our study is the relatively small sample size, which may limit the generalization of the results. However, we need to understand that it may not be feasible to generate a large sample of patients in such cohorts. Also, our study has other several limitations including the use of different atherectomy devices, with different size and number of burr. Moreover, severely reduced LVEF was defined differently in the included studies. In addition, the studies were non-randomized, which can have inherent selection bias. Other main limitation is that the outcomes were not adjusted for different variables. Despite all these limitations, we believe that the current review adds to the understanding of clinical outcomes in patients with reduced LVEF with severely calcified lesions with the use of atherectomy.

## Conclusion

Our meta-analysis suggested that the patients with severely reduced LVEF when using atherectomy devices experienced higher risk of clinical outcomes in the terms of all-cause mortality and cardiac mortality. As we know that the patients with severely reduced LVEF are inherently at increased risk of adverse clinical outcomes, this information should be considered hypothesis generating and utilized while discussing the risks and benefits of atherectomy in such high risk patients. Future studies should focus on the comparison of outcomes of different atherectomy devices in such patients. Adjusting for the inherent mortality risk posed by left ventricular dysfunction may be a strategy while designing a study.

## Data availability statement

The original contributions presented in the study are included in the article/Supplementary material, further inquiries can be directed to the corresponding author/s.

## Author contributions

WA, MRM, KD, and AA-A commenced the idea of this research. WA, MRM, AA-A, QR, MI, MA, JS, and NE drafted this manuscript. WA and MRM collected the data of this research. WA, MRM, and AA-A analyzed these data. TP, IB-D, KD, WA, and MRM critically reviewed this manuscript. All authors designed this research and approved the final manuscript. WA and KD supervised this research. All authors contributed to the article and approved the submitted version.

## Conflict of interest

The authors declare that the research was conducted in the absence of any commercial or financial relationships that could be construed as a potential conflict of interest.

## Publisher's note

All claims expressed in this article are solely those of the authors and do not necessarily represent those of their affiliated organizations, or those of the publisher, the editors and the reviewers. Any product that may be evaluated in this article, or claim that may be made by its manufacturer, is not guaranteed or endorsed by the publisher.

## Supplementary material

Supplemental Figure 1 Funnel plot of the in-hospital and long term outcomes. MI, myocardial infarction; TVR, target vessel revascularization.

Supplemental Table 1 Search Strategy.

Supplemental Table 2 Risk of bias assessment.

Supplemental Table 3 Summarizing the definition of in-hospital myocardial infarction in each included study.

## Abbreviations

PCI: Percutaneous coronary intervention
LVEF: Left ventricular ejection fraction
CAC: Coronary artery calcification
CABG: Coronary artery bypass grafting.
