# Supplementary figures and images for: Outcomes of atherectomy in treating severely calcified coronary lesions in patients with reduced left ventricular ejection fraction: A systematic review and meta-analysis
Source: Front Cardiovasc Med. 2022 Sep 20;9:946027. doi: 10.3389/fcvm.2022.946027 (PMC9530054; doi:10.3389/fcvm.2022.946027)

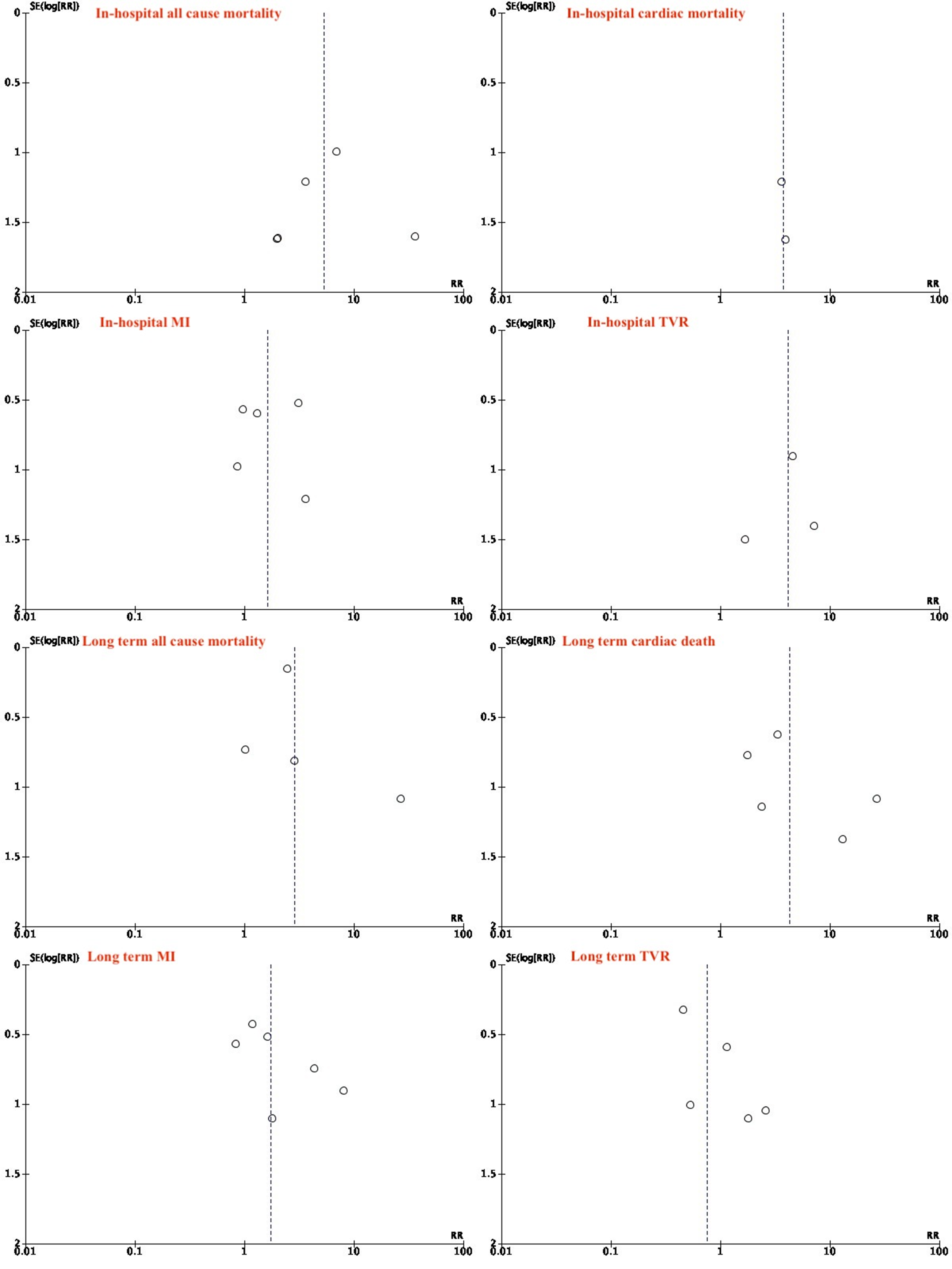

Supplement: Supplemental Figure 1 — Funnel plot of the in-hospital and long term outcomes. MI, myocardial infarction; TVR, target vessel revascularization. [file Image_1.tiff]
