# Supplementary material for: Outcomes of atherectomy in treating severely calcified coronary lesions in patients with reduced left ventricular ejection fraction: A systematic review and meta-analysis
Source: Front Cardiovasc Med. 2022 Sep 20;9:946027. doi: 10.3389/fcvm.2022.946027 (PMC9530054; doi:10.3389/fcvm.2022.946027)
Supplement: Supplemental Table 1 — Search Strategy. [file Table_1.docx]

**Supplementary Table 1: Search Strategy**

| **Pubmed** | | | |
| --- | --- | --- | --- |
| **No.** | **Search terms** | | **Number of items** |
| 2 | Atherectomy | (atherectomy[Title/Abstract]) OR (atherectomy[MeSH Terms]) | 4,404 |
| 3 | Heart failure | ("atherectomy"[Title/Abstract] OR "atherectomy"[MeSH Terms]) AND ("HFrEF"[All Fields] OR "left ventricular systolic dysfunction"[Title/Abstract] OR "impaired left ventricular ejection fraction"[Title/Abstract] OR "reduced left ventricular ejection fraction"[Title/Abstract] OR "heart failure with reduced ejection fraction"[Title/Abstract] OR "heart failure"[Title/Abstract] OR "systolic dysfunction"[Title/Abstract]) | 194,440 |
| 4 | Combined search | #1 AND #2 | 59 |
| **Cochrane Library** | | | |
| 1 | Atherectomy AND heart failure | Atherectomy [Title/Abstract]  AND left ventricular systolic dysfunction [Title/Abstract] | 23 |
| **ClinicalTrials.gov** | | | |
| 1 | Atherectomy AND heart failure | Atherectomy [All studies] AND heart failure [All studies] | 27 |
