# Supplementary material for: Outcomes of atherectomy in treating severely calcified coronary lesions in patients with reduced left ventricular ejection fraction: A systematic review and meta-analysis
Source: Front Cardiovasc Med. 2022 Sep 20;9:946027. doi: 10.3389/fcvm.2022.946027 (PMC9530054; doi:10.3389/fcvm.2022.946027)
Supplement: Supplemental Table 2 — Risk of bias assessment. [file Table_2.docx]

| The Newcastle-Ottawa Scale for assessing the quality of nonrandomized studies in the meta-analysis. | | | | | | | | | |
| --- | --- | --- | --- | --- | --- | --- | --- | --- | --- |
|  | Selection | | | | | Outcome | | | |
| Study | Representativeness of the exposed cohort | Selection of the non-exposed cohort | Ascertainment of exposure | Outcome not present at baseline | Comparability of the cohort | Assessment of outcome | Enough follow up duration | Adequate follow-up | Total score |
| Lee 2017 | NA | * | * | * | * | * | * | * | 7 |
| Shlofmitz  2017 | NA | * | * | * | NA | * | NA | * | 5 |
| Watanabe  2018 | NA | * | * | * | NA | * | NA | * | 5 |
| Whiteside 2018 | NA | * | * | * | NA | * | * | * | 6 |
| Zhang 2019 | NA | * | * | * | * | * | * | * | 7 |
| Mankerious 2019 | NA | * | * | * | * | * | * | * | 7 |
| Yoshida 2020 | NA | * | * | * | * | * | * | * | 7 |

Each asterisk represents one star in the Newcastle-Ottawa Scaling System (NOS). The maximum stars are 2 for comparability and 1 for all other categories. Each star counts towards the total score. Score of 5 to 6 considered as moderate quality and 7 to 9 as high quality.

Abbreviation: NA: not available.
