# Supplementary material for: Outcomes of atherectomy in treating severely calcified coronary lesions in patients with reduced left ventricular ejection fraction: A systematic review and meta-analysis
Source: Front Cardiovasc Med. 2022 Sep 20;9:946027. doi: 10.3389/fcvm.2022.946027 (PMC9530054; doi:10.3389/fcvm.2022.946027)
Supplement: Supplemental Table 3 — Summarizing the definition of in-hospital myocardial infarction in each included study. [file Table_3.docx]

| **Study** | **Definition of in-hospital myocardial infarction (MI)** |
| --- | --- |
| **2017 Lee et al.** | Increased creatine kinase-myocardial band level >3x the upper limit of normal with or without a new pathologic Q-wave. |
| **2017 shlofmitz et al.** | Recurrent symptoms with new ST-segment elevation or re-elevation of cardiac markers to at least twice the upper limit of normal. |
| **2018 Watanabe et al.** | Creatine kinase muscle-brain type elevation greater than three-fold the upper normal level with Q waves or without Q waves on the electrocardiogram 24 hours after surgery. |
| **2018 whiteside et al.** | Development of new ST-segment elevation and/or a rise in cardiac biomarkers above the previously documented value in addition to ischemic symptoms |
| **2019 Mankerious et al.** | Defined according to the third universal definition of MI |
| **2019 Zhang et al.** | Elevation of the cTn level to >2 times the upper limit of the reference range and recurrent symptoms with or without new ST-segment changes. |
| **2020 Yoshida et al.** | Presence of pathological and new Q waves on an electrocardiogram, or an in- crease in the creatine kinase myocardial-band level to more than two times the upper limit of the normal range |

Supplemental table 3: Definition of in-hospital myocardial infarction in each included study.
